# The Making of the Modern Practitioner

**Published:** 1912-01-27

**Authors:** 


### THE MAKING OF THE MODERN PRACTITIONER.

### Degrees and Diplomas.—I.

The British medical practitioner as he exists to-day is a person who has been able to pick and choose between a very large number of examining corporations in his student days. Each of these bodies has the power to confer upon him its own particular style or title; this enables him to use the initials thereof as a suffix to his name, and to claim as a right admission to the Medical Register on payment of the statutory fee to the General Medical Council. About the various degrees, licences, and diplomas which carry with them this privilege there is among the public at large a very excusable amount of ignorance, and also of entire misconception; and even in the ranks of the medical profession a great deal of disagreement and controversy. It is worth while, therefore, to state a few of the admitted facts which the public, and especially the hospital public, ought to know; while touching also very slightly upon those points which have been and still are the cause of heart-burning to individual members of the profession.

### Graduating v. Registration.

To begin with, then, as has already been indicated, the actual registering authority is the General Medical Council. Registration confers certain important privileges, such as the right to sue for fees, the right to hold various appointments which are not open to the unregistered, the right to sign various documents such as death certificates, and others. It is, as will be comprehended, a separate process altogether from the acquisition of the degree or diploma which is thus registered. It follows also that to strike off the Register anyone who is already on it is the function of the General Medical Council alone, which can do this for sufficient cause shown, but cannot cancel the degree or diploma of the person thus struck off. Similarly this latter process is purely a domestic matter reserved for the constituted authority of the corporation which has granted its registrable qualification to the *alumnus*.

It is true that the Medical Council is charged with the duty of supervising the separate examining corporations, and of insisting upon such a standard of stringency as may be deemed necessary; this duty the Medical Council very strictly carries out. The duty thus imposed by the State is really a tacit recognition of the principle of one uniform portal to the medical profession, though, with the usual British love of half-measures, it is not pushed to its logical conclusion, State examinations for all medical students of the United Kingdom.

So much for the method of registration. The number of bodies which are competent to grant a registrable qualification is fairly large; and it is in respect of their various distinguishing titles that the public very naturally gets so often confused. Many schemes have been propounded at different times by would-be reformers of the present make-shift system, but none has been within measurable distance of adoption.

### Examinations v. Experience.

One reason for this is doubtless that examinations and the titular distinctions which depend on them are of less importance in the eyes of the general public than tact, clinical acumen, and experience. A man may have passed with the highest possible distinction those examinations which are the best esteemed by the laity (and professional colleagues); but if he be a mere bookworm or have a tactless manner, he will take second place to a man less capable of outwitting an examiner but more conversant with human nature and everyday problems of disease. Examinations, in fact, are no test at all of common-sense, without which no doctor is worth his salt.

At the same time, degrees and diplomas are very, very far from negligible. For one thing they are of the most vital importance whenever there is any public post or office to be filled by competition. Here they have very often an altogether fictitious and exaggerated value, because they are in many cases the only criterion which a non-medical selection committee possesses of discriminating between one candidate and another. Let no student, therefore, imagine that one qualification will be as good to him as another; for he never knows when he may have to submit those he possesses to the scrutiny of men who have no means of assessing those real signs of efficiency which could be taken into account by his professional brethren.

Further, there are certain examinations which are earmarked, as it were, for special purposes; and as such have a special value. The best example is the Diploma in Public Health (D.P.H.), which is now indispensable for anyone who aspires to whole-time employment in the organised sanitary service of the community. Yet be it remarked also that the D.P.H. is not of itself a registrable qualification; the would-be public health officer must pass first some one of the ordinary qualifying examinations.

There are also signs of the times which indicate the future possible extension of the system thus initiated in connection with public health. Thus an attempt has been made by the ophthalmic surgeons to set up a standard of their own, whose attainment shall hall-mark some future generation of specialists in this department. At the University of Oxford a Diplomate in Ophthalmology (D.O.) was instituted a year or two ago; and although the matter has not got very far yet, the leaven of this example may work in time. So, too, in tropical medicine special tests of attainment have been created by several of the educational authorities; and the tacit recognition of the D.T.M. by the Colonial Office is bound to encourage those who urge the extension of the system to all special departments of practice.

